# ImmunoAIzer: A Deep Learning-Based Computational Framework to Characterize Cell Distribution and Gene Mutation in Tumor Microenvironment

**DOI:** 10.3390/cancers13071659

**Published:** 2021-04-01

**Authors:** Chang Bian, Yu Wang, Zhihao Lu, Yu An, Hanfan Wang, Lingxin Kong, Yang Du, Jie Tian

**Affiliations:** 1 CAS Key Laboratory of Molecular Imaging, The State Key Laboratory of Management and Control for Complex Systems, Institute of Automation, Chinese Academy of Sciences, Beijing 100190, China; bianchang2018@ia.ac.cn (C.B.); wangyu2019@ia.ac.cn (Y.W.); yuan1989@buaa.edu.cn (Y.A.); wanghanfan2020@163.com (H.W.); konglingxin17@mails.ucas.ac.cn (L.K.); 2 School of Artificial Intelligence, University of Chinese Academy of Sciences, Beijing 100049, China; 3 Department of Gastrointestinal Oncology, Key Laboratory of Carcinogenesis and Translational Research (Ministry of Education), Peking University Cancer Hospital & Institute, Beijing 100142, China; zhihaolupku@bjmu.edu.cn; 4 Beijing Advanced Innovation Center for Big Data-Based Precision Medicine, School of Medicine Science and Engineering, Beihang University, Beijing 100191, China; 5 School of Life Science and Technology, Xidian University, Xi’an 710071, China

**Keywords:** deep learning, cell distribution, biomarker, tumor gene mutation, tumor microenvironment (TME), semi-supervised learning, hematoxylin and eosin (H&E)

## Abstract

**Simple Summary:**

A comprehensive evaluation of immune cell distribution in the tumor microenvironment (TME) and tumor gene mutation status may contribute to therapeutic optimization of cancer patients. In this study, we aimed to demonstrate that deep learning (DL)-based computational frameworks have remarkable potential as a tool to analyze the spatial distribution of immune cells and cancer cells in TME and detect tumor gene mutations. TME analysis can benefit from the computational framework, mainly due to its efficiency and low cost. Cells distribution in TME and tumor gene mutation status can be characterized accurately and efficiently. This may lead to a reduced working load of pathologists and may result in an improved and more standardized workflow.

**Abstract:**

Spatial distribution of tumor infiltrating lymphocytes (TILs) and cancer cells in the tumor microenvironment (TME) along with tumor gene mutation status are of vital importance to the guidance of cancer immunotherapy and prognoses. In this work, we developed a deep learning-based computational framework, termed ImmunoAIzer, which involves: (1) the implementation of a semi-supervised strategy to train a cellular biomarker distribution prediction network (CBDPN) to make predictions of spatial distributions of CD3, CD20, PanCK, and DAPI biomarkers in the tumor microenvironment with an accuracy of 90.4%; (2) using CBDPN to select tumor areas on hematoxylin and eosin (H&E) staining tissue slides and training a multilabel tumor gene mutation detection network (TGMDN), which can detect APC, KRAS, and TP53 mutations with area-under-the-curve (AUC) values of 0.76, 0.77, and 0.79. These findings suggest that ImmunoAIzer could provide comprehensive information of cell distribution and tumor gene mutation status of colon cancer patients efficiently and less costly; hence, it could serve as an effective auxiliary tool for the guidance of immunotherapy and prognoses. The method is also generalizable and has the potential to be extended for application to other types of cancers other than colon cancer.

## 1. Introduction

The tumor microenvironment (TME) is the internal environment in which tumor grows [1,2,3]. The understanding of TME, especially the spatial distribution of tumor infiltrating lymphocytes (TILs) within TME, can be of great importance to offer guidance for immunotherapy [4,5,6,7] and evaluating prognosis of cancer [8]. Moreover, tumor gene mutation status is also of vital importance for the prediction of drug efficacy [9], disease-free survival [10], and immunotherapy response [11,12]. As such, a comprehensive evaluation of immune cell distribution in TMEs and tumor gene mutation status may offer guidance for patient selection and therapeutic optimization.

Currently, the histological analysis of hematoxylin and eosin (H&E)-stained tissue is considered the gold standard for pathologic diagnoses. Multiplexed immunohistochemistry (mIHC) techniques can help pathologists interpret clinically relevant cellular subtypes and biomolecules, and are widely applied in TME analysis [13,14,15]. The mIHC-based methods allow simultaneous display of the cellular expressions of several biomarkers on tissue slides; thus, they offer more comprehensive insight into disease heterogeneity. However, mIHC has certain limitations [16]. First, the multiplexed staining method is both time- and cost-consuming. Second, spectrum overlapping can affect the accuracy of the staining results. Third, a professional pathologist is often required to interpret mIHC-based images; thus, the interpretation is subject to individual subjectivity. Therefore, there is an urgent need to develop an alternative and more efficient method to complement the existing mIHC approach to characterize different cell types in the TME.

A computational framework may provide a more comprehensive, accurate, and objective method to assist in therapeutic optimization [17]. The application of deep learning (DL) in medical image processing, especially histological image analysis, has been shown to extract valuable information that is imperceptible to humans [18,19,20]. Recent advances in DL and digital pathology have made it possible to derive fluorescence images from transmitted light images of unlabeled fixed or live biological samples [21], thus demonstrating the capability and reliability of DL methodology as a tool to facilitate biomarker prediction tasks. Burlingame et al. introduced an adversarial deep learning method that they used to infer fluorescence images based on the H&E-stained tissue from a patient with pancreatic ductal adenocarcinoma [22], further verifying the practicability of DL-based methods as a type of prediction tool for complex human tissues. Moreover, previous research has also suggested that a DL neural network can detect gene mutations based on H&E images [23], demonstrating the potential application of DL-based methods in the field of pathological image analysis. Hence, we hypothesized that DL may facilitate the evaluation of immune cell and cancer cell distribution in TMEs by predicting the cellular biomarker distribution, meanwhile offer guidance for tumor gene mutation analysis.

Despite the advancements of DL-based methods in medical image analysis, most existing DL-based methods are fully supervised and require a large amount of labeled data for training to achieve an acceptable level of performance, especially when they are applied to pixel-level segmentation tasks. However, the annotation of medical image data can be very laborious and time-consuming, and it often requires professional pathologists to ensure accuracy [24]. Thus, it would be very beneficial to develop semi-supervised DL- based computational methods [25] to leverage unlabeled image data to increase the efficiency. Moreover, The Cancer Genome Atlas (TCGA) dataset contains a massive amount of H&E staining data from cancer patients; however, it remains underutilized and can be useful for a semi-supervised approach.

**Figure 1:**
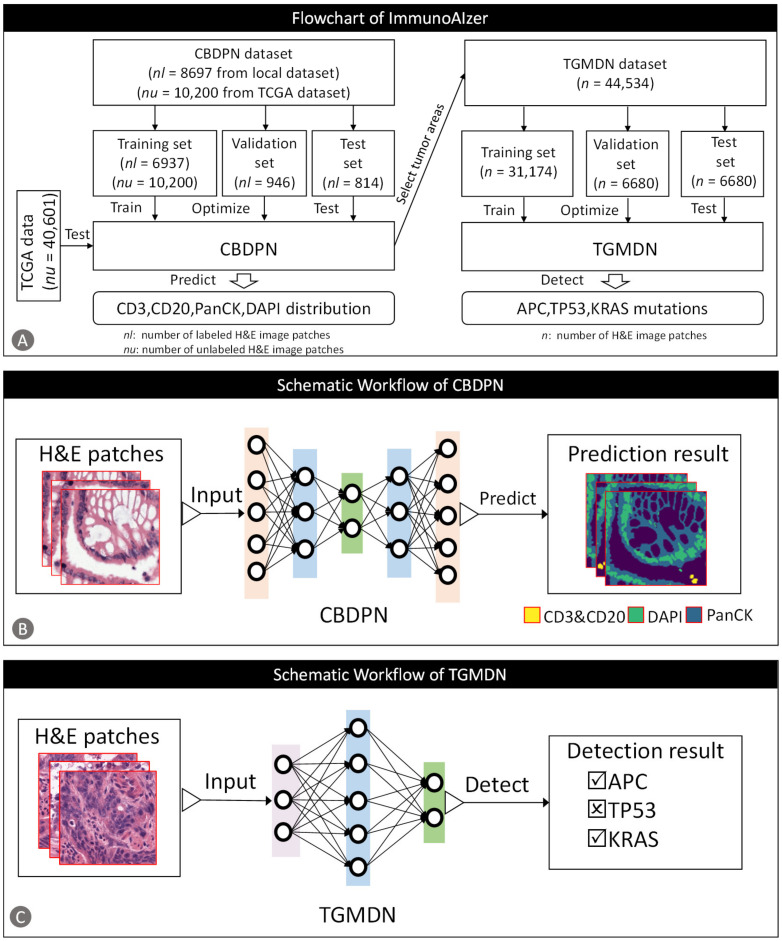
Study protocol workflow. (**A**) ImmunoAIzer includes: a cellular biomarker distribution prediction network (CBDPN) and a tumor gene mutation detection network (TGMDN). (**B**) CBDPN takes H&E image patches as input and makes predictions of the spatial distribution of CD3 and CD20, pan-cytokeratin (PanCK) and DAPI in TME. (**C**) TGMDN takes H&E image patches as input and detects adenomatous polyposis coli gene (APC), tumor protein P53 gene (TP53), and kirsten rat sarcoma viral oncogene (KRAS) mutations. (TCGA: The Cancer Genome Atlas; H&E: hematoxylin and eosin)

In this study, we proposed a computational framework, termed ImmunoAIzer, which can use H&E images to provide comprehensive and valuable information for pathologists to aid TME analysis. ImmunoAIzer contains two components: (1) a semi-supervised cellular biomarker distribution prediction network (CBDPN) which can make predictions of the spatial distribution of CD3, CD20, PanCK, and DAPI on H&E images; (2) a tumor gene mutation detection network (TGMDN), which can detect APC, TP53, and KRAS gene mutations from H&E images. The flowchart of ImmunoAIzer is described in Figure 1. In general, our proposed computational framework can reveal TILs and cancer cells distribution in TME, and facilitate tumor gene mutation assessment. These factors allow the framework to be used as an effective auxiliary tool for the comprehensive evaluation of TME and as a reference for the therapeutic optimization. Furthermore, our method can be extended for application to different cancer types.

## 2. Materials and Methods

### 2.1. Dataset Establishment

The data we used in our work includes two parts: (1) data used for CBDPN training and validation, and (2) data used for TGMDN training and validation.

For the CBDPN, the dataset included two types of data: (1) pixel-level labeled H&E image data of 8697 H&E image patches and their corresponding label masks of 8 colon cancer patients from Peking University Cancer Hospital and Institute, and (2) unlabeled image data of 50,801 H&E image patches of 60 colon cancer patients acquired from the TCGA dataset, Colon Adenocarcinoma (COAD) project. For the tissue samples acquired from Peking University Cancer Hospital and Institute, the H&E and mIHC stains were conducted. PanCK was used to characterize tumor cells, and CD3 and CD20 were used to characterize TILs [26], and DAPI for nuclei. The staining and scanning protocols are described in Appendix A. First, in order to facilitate annotation, a registration procedure was performed after acquisition of the H&E and mIHC images to ensure that the H&E and mIHC images were spatially registered. Second, the registered H&E and mIHC whole slide images (WSI) were tiled into 512 × 512-pixel patches, and were pixel-level annotated by 3 board certified pathologists. Third, a color normalization [27,28] procedure was conducted to improve the image quality. Finally, we conducted a data augmentation procedure to obtain 8697 H&E image patches of which the training, validation, and testing image data ratio was set to be approximately 8:1:1, yielding 6937 image patches in the training set, 946 image patches in the validation set, and 814 images in the test set. The whole data processing pipeline is illustrated in Figure S1. The registration procedure and other post-processing details are described in Appendix B. Additionally, for the unlabeled image data from the TCGA dataset, the WSIs of 60 patients were tiled into 512 × 512-pixel patches, yielding 50,801 image patches in total, among which 10,200 image patches of 35 patients with background proportion below 50% were utilized for semi-supervised training, and 40,601 image patches of the other 25 patients were utilized for performance validation.

For the TGMDN, H&E-stained WSIs from 446 colon cancer patients with gene mutations were selected from the TCGA dataset COAD project. In order to make sure that the dataset contains sufficient images with mutations, we chose those which were mutated in at least 10% of the tumor areas. The WSIs were tiled into 512 × 512-pixel patches. For each H&E-stained slide, only patches predicted through our CBDPN to have a PanCK-positive area of more than 50% were selected for implementation. This condition was necessary to choose the images of the tumor area. Finally, we used CBDPN to select 44,534 image patches from 339 patients for the task. The training, validation, and testing image ratio was set to be approximately 14:3:3, respectively, yielding 31,174 H&E image patches in the training set, 6680 H&E images in the validation set, and 6680 H&E images in the test set. All of the H&E patches were intensity-normalized [27,28] prior to training.

### 2.2. CBDPN Training and Validation

#### 2.2.1. Semi-Supervised Mechanism

**Figure 2:**
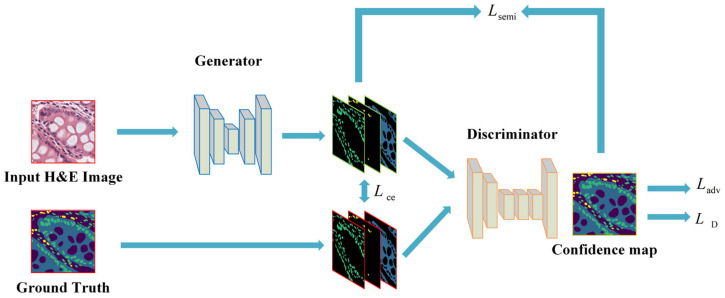
Semi-supervised structure for the ImmunoAIzer cellular biomarker prediction networks. Note that the generator and discriminator structure in this figure is only schematic, a detailed introduction about the generator structures will be given in the following sections.

In order to tackle the problem of insufficient labeled data and the heavy burden of annotation work, our semi-supervised biomarker distribution prediction network utilizes an adversarial learning paradigm [29], the structure of which consists of a generator and a discriminator. In order to better extract useful features from pathological images and optimize performance, the generator was structured to have an encoder–decoder form. The generator can be any form of segmentation network which takes in the original H&E image and outputs the class probability maps and the discriminator is organized the same as [29]. Note that the generator can also be used as an independent, fully supervised biomarker distribution prediction network. The semi-supervised network structure is illustrated in Figure 2.

Given an input H&E image, Xn, of size H × W × 3, the segmentation network of the generator is denoted as $S\left(\cdot \right)$; the predicted probability map, $S\left(Xn\right),$ of size H × W × C where C is the category number; the applied categories were background, PanCK-positive area, CD3 and CD20-positive area, and DAPI. Note that CD3 and CD20 positive cells were regarded as one class as TILs in our study [26]. The discriminator is denoted as $D\left(\cdot \right)$, which takes in a probability map of size H × W × C and outputs a confidence map of size H × W × 1. In this study, we applied two possible inputs to the discriminator: (1) a biomarker distribution prediction, $S\left(Xn\right),$ and (2) a one-hot-encoded mask vector, Yn. Each pixel p of the discriminator output map indicates whether the pixel was sampled from the ground truth labeled by the pathologists *p* = 1 or from the predictions made by our segmentation network *p* = 0. In this way, we can leverage both labeled data and unlabeled data to train the network.

The generator was trained by minimizing the multitask loss function:

(1) $$L_{seg}=\;L_{ce}\;+\lambda_{adv}L_{adv}\;+\lambda_{semi}L_{semi},$$

where $L_{ce}$, $L_{adv}$, and $L_{semi}$ represent multiclass cross-entropy loss, adversarial loss, and semi-supervised loss, respectively. In (1), $\lambda_{adv}$ and $\lambda_{semi}$ are two weighted parameters. As the network is trained on the labeled data, the cross-entropy loss is calculated as:

(2) $$L_{ce}=-\underset{h,w}{\sum }\underset{c\in C}{\sum }Y_{n}^{\left(h,w,c\right)}\log(S{\left(X_{n}\right)}^{\left(h,w,c\right)}).$$

The adversarial learning strategy is applied to maximize loss $L_{adv}$, as follows:

(3) $$L_{adv}=-\sum_{h,w}\log\left(D{\left(S\left(X_{n}\right)\right)}^{\left(h,w\right)}\right).$$

This loss function was applied to fool the discriminator by maximizing the probability of the predicted results being generated from the ground-truth label mask.

The discriminator was trained by applying the spatial cross-entropy loss of the two causes; this was achieved by performing the following operation:

(4) $$L_{D}=-\sum_{h,w}\left(1-y_{n}\right)\log(1-D{\left(S\left(X_{n}\right)\right)}^{\left(h,w\right)})+y_{n}\log\left(D{\left(Y_{n}\right)}^{\left(h,w\right)}\right),$$

under the condition that the input is given as the output of the generator, $y_{n}=0$; alternatively, under the condition that the sample input was taken from the ground truth labels, $y_{n}=1$. In (4), $D(S{\left(X_{n}\right)}^{\left(h,w\right)}$ represents the confidence map of X at coordinates $\left(h,w\right)$; $D{\left(Y_{n}\right)}^{\left(h,w\right)}$ is similarly defined.

Note that $L_{ce}$ was not applied in the case of unlabeled data because there was no ground-truth mask. To prevent the prediction from being overcorrected by the adversarial loss, we applied a smaller $\lambda_{adv}$ to the loss function. The segmentation network outputs an initial prediction, $S\left(X_{n}\right)$, and the discriminator generates a confidence map, $D\left(S\left(X_{n}\right)\right)$, to indicate the regions that are sufficiently close to the ground-truth biomarker distribution masks. A threshold was applied to establish the trustworthy region, i.e., the region that can be used as the label to reasonably train the segmentation network in a self-taught manner. The resulting semi-supervised loss is defined as:

(5) $$L_{semi}=-\underset{h,w}{\sum }\underset{c\in C}{\sum }I\left(D{\left(S\left(X_{n}\right)\right)}^{\left(h,w\right)}>T_{semi}\right)\cdot {\hat{Y}}_{n}^{\left(h,w,c\right)}\log\left(S{\left(X_{n}\right)}^{\left(h,w,c\right)}\right).$$

In (5), $I\left(\cdot \right)$ is the indicator function, $T_{semi}$ is the threshold used to select the trustworthy region, ${\hat{Y}}_{n}$ is the one-hot-encoded ground truth, and ${\hat{Y}}_{n}^{\left(h,w,c\right)}=1$ if $c=argmax_{c}S{\left(X_{n}\right)}^{\left(h,w,c\right)}$.

#### 2.2.2. CBPDN Structure

**Figure 3:**
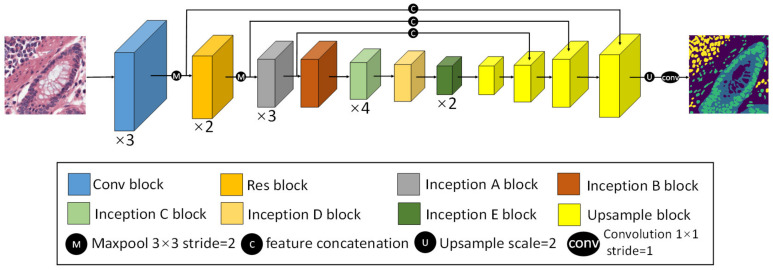
CBPDN structure to predict biomarker distributions. Inception blocks A to D are a series of convolutional modules at various resolutions which are used extract features at different levels, and Inception block E is used to promote high dimensional representations to boost prediction performance. Details of the module structure are described in Figure S3.

In order to better extract features from pathological images, we equipped CBDPN with a combination of residual blocks [30], and Inception V3 [31] architecture as the backbone. The Inception V3 architecture comprises inception units, with each inception unit consisting of several nonlinear convolutional modules at various resolutions, which makes it very useful for pathology-related image-processing tasks. We added two residual blocks after the first max-pooling layer to improve the feature-extraction performance. The network structure is illustrated in Figure 3. In addition, the structure details of each block are described in Figure S3. CBPDN can be used as the generator in the semi supervised mechanism described in Section 2.2.1 and it can also be used independently as a fully supervised segmentation network.

For the discriminator, it consists of 5 convolution layers with 4 × 4 kernel in the stride of 2. The channels of these layers are 64, 128, 256, 512, and 1, respectively. Each convolution layer is followed by batch normalization and Leaky ReLU. An up-sampling layer is added to the last layer to rescale the output to the size of the input map to transform the model into a fully convolutional network.

#### 2.2.3. Test Set Validation

For CBPDN performance evaluation, first, we applied the dataset with labeled images in independent training and testing experiments using CBDPN and three conventional segmentation networks, i.e., UNet [32], DeepLabV3 [33], and DeepLabV3+ [34] in the fully supervised manner, to prove the superiority of our proposed approach in making predictions of the biomarker distribution. Second, to prove the effectiveness of the semi-supervised mechanism, we incorporated CBDPN and the other three networks with the semi-supervised structure. It is worth mentioning that in this case, the segmentation networks were used as the generator of the entire structure described in Figure 2. Further, 10,200 unlabeled H&E image patches were added in the training phase. In addition, 814 H&E images with labels were used for validation.

The metrics used to evaluate the performance of the biomarker distribution prediction network were the dice coefficient, and intersection-over-union (IoU), precision, recall. We used TP, FP, TN, and FN to denote the true-positive, false-positive, true-negative, and false-negative predictions, respectively. In addition, x,y are used to represent the prediction result and the ground truth map, respectively. Further, k denotes the total number of classes. The IoU and Dice metrics are defined as:

(6) $$IoU=\frac{\sum_{i=1}^{k}\frac{\left|x_{i}\cap y_{i}\right|}{\left|x_{i}\cup y_{i}\right|}}{k},$$

(7) $$dice=\frac{2\cdot \sum_{i=1}^{k}\frac{\left|x_{i}\cap y_{i}\right|}{\left|x_{i}\right|+\left|y_{i}\right|}}{k},$$

which measure the overlap between prediction x and ground truth y.

The precision score was calculated as follows:

(8) $$precision=\frac{\sum_{i=1}^{k}\frac{TP_{i}}{TP_{i}+FP_{i}}}{k}.$$

The recall score was calculated as follows:

(9) $$recall=\frac{\sum_{i=1}^{k}\frac{TP_{i}}{TP_{i}+FN_{i}}}{k}.$$

For each patch, the prediction result and the ground truth label were compressed into one channel and each pixel was labeled to represent one specific marker. For the prediction result, the label with the highest probability of each pixel point being predicted is considered to be the final marker classification of that pixel. In addition, the final metrics were calculated on pixel-level by averaging the metric of each prediction class.

#### 2.2.4. TCGA Dataset Validation

In order to prove the robustness and generalization ability of CBDPN, we used 43,601 H&E image patches of 25 colon cancer patients from the TCGA dataset for further validation. Because the corresponding mIHC staining images of the TCGA WSIs were not available, we were unable to make label masks and calculate the accuracy and dice coefficient. Instead, we calculated the PanCK-positive cell fraction and CD3- and CD20-positive cell fractions given by our optimal model, and then compared the results to the cell fractions determined based on the molecular information available in the TCGA dataset. The TCGA benchmark cell fraction was obtained from molecular genomics assays based on DNA methylation arrays and RNA sequencing data. This is a reasonable approach because PanCK is typically used to characterize tumor cells, and CD3 and CD20 are used to characterize TILs [26].

#### 2.2.5. CBDPN-Based Cell Quantification

In order to prove the utility of CBDPN in clinical scenarios, considering programmed cell death protein 1 (PD-1) is a crucial biomarker for TILs [35,36,37], we also tested the ability of CBDPN to aid quantification of PD-1-expressing cells in TME in the presence of anti-PD-1 immunofluorescence staining. We first acquired H&E images and anti-PD-1 immunofluorescence images of the same tissue. We used CBDPN to acquire cell-specific biomarker distribution in TME, and then we used ImageJ software [38] to preprocess the anti-PD-1 immunofluorescence image to determine PD-1-positive cell distribution. Finally, we merge the cell-specific biomarker distribution generated by CBDPN and the PD-1-positive cell distribution image to gain visualization of the spatial distribution of PD-1-expressing TILs. The quantification can then be achieved by using ImageJ software (Version 1.52p, Fiji-64bit).

In clinical settings, pathologists often use mIHC staining images and Inform software [39] (PerkinElmer, Waltham, MA, USA) to characterize and quantify the PD-1 expression in TME. Inform software (Version 2.4) uses mIHC image as input to experimentally measure cell proportion. The basic workflow is that Inform software first localizes nuclei distribution on the mIHC WSI and then detects the type of fluorescence marker closest to each nucleus to determine the biomarker expression status at cellular level.

To validate the accuracy of our CBDPN-based cell quantification method, we compared our results with the results calculated using the commercial Inform software based on mIHC WSIs of the same tissues.

### 2.3. TGMDN Training and Validation

#### 2.3.1. TGMDN Structure

**Figure 4:**
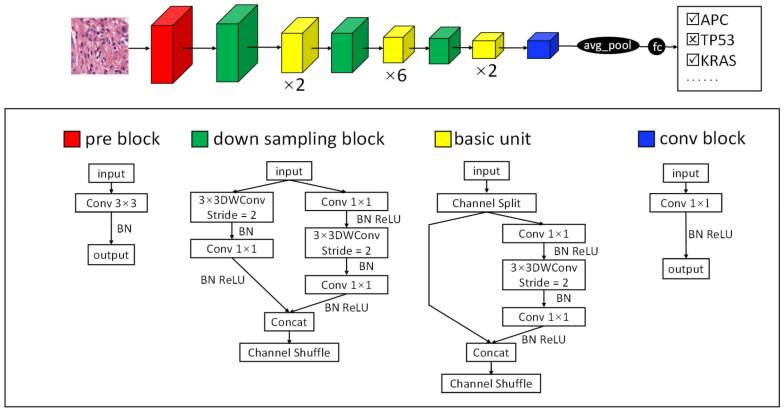
TGMDN structure to detect tumor gene mutations.

For the TGMDN, we used the ShuffleNet V2 [40] network to conduct the multilabel training process to detect APC, TP53, and KRAS mutations in colon cancer patients. ShuffleNet V2 was selected because it has demonstrated good performance on gene mutation detection tasks [41]. The network learned the features of each of the three mutations, with each mutation classification corresponding to an independent binary classification. We implemented a binary cross-entropy loss function and sigmoid layer to replace the softmax layer; this allowed each H&E patch to be associated with several binary labels. The entire network structure is illustrated in Figure 4.

#### 2.3.2. TGMDN Evaluation

To test the performance of the TGMDN, we tested the detection accuracy on the test set containing 6680 H&E image patches from TCGA dataset and calculated the receiver operating characteristic (ROC) of these three mutations.

Moreover, in order to better understand the gene mutation association, we also employed a t-SNE method [42], utilizing the values of the last fully connected layer of our TGMDN as input, to visualize how the mutations and patches were organized in the multidimensional space of the network.

### 2.4. Implementation Details

Our method is implemented based on the PyTorch DL framework using Python [43]. Two pieces of TESLA V100 GPUs were employed to accelerate the training. For semi-supervised CBDPN training, the batch size was set as 8 and the initial learning rate was set as 0.001 for the generator, and 10^−6^ for the discriminator. $\lambda_{semi}$ was set as 0.1; $\lambda_{adv}$ was set as 0.01 and 0.001 for labeled and unlabeled data, respectively, and $T_{semi}$ was set as 0.5. To train the predictive network, we use the Adam optimizer method, where the momentum is 0.9, and the weight decay is 10^−8^. We first train the generator for 10,000 steps and then train the generator and discriminator simultaneously for 300,000 steps. We start the semi-supervised training after training 100,000 steps with labeled data. For TGMDN training, we implemented the RMSprop optimization to train the network for 100 epochs with batch size as 16. We set the momentum as 0.9, and the weight decay as 10^−8^. The initial learning rate is 0.01. Statistical analysis is conducted using Prism 6 software.

## 3. Results

### 3.1. CBDPN for the Prediction of Cellular Biomarker Distribution in TME

#### 3.1.1. Fully-Supervised Experiment Results

We used CBDPN, UNet, DeeplabV3, and DeeplabV3+ to train the biomarker prediction model. The results on our test set (including 814 H&E image patches) are summarized in Table 1. As observed, our CBDPN yielded optimal results in the fully supervised experiments. Our proposed network achieved the highest scores for accuracy, precision, IoU, and dice among the four networks. Although CBDPN did not achieve the highest score for recall, it achieved the best dice coefficient, which provides information on the trade-off between precision and recall. The results prove the effectiveness and reliability of CBDPN in predicting cell biomarker distributions.

#### 3.1.2. Semi-Supervised Experiment Results

**Figure 5:**
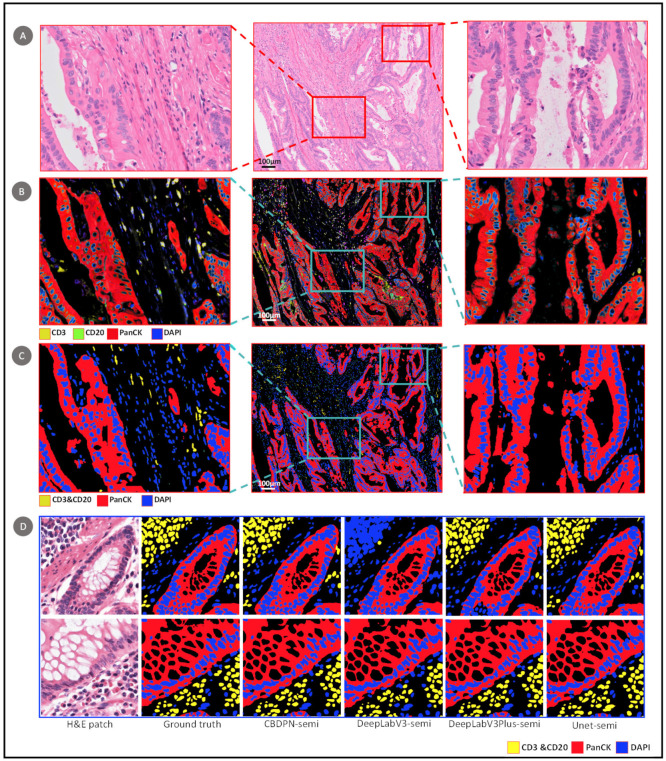
Cellular biomarker prediction results. (**A**) H&E-stained colon cancer slides: the middle image shows an H&E-stained slide (~5500 × 4000 pixels). (The left and right images show enlarged views.) (**B**) mIHC image of the same tissue sample. (**C**) Predictions of our proposed biomarker prediction network. (**D**) 512 × 512 patch-wise results for our proposed method and UNet, DeepLabV3 and DeepLabV3+ under the semi-supervised structure.

We incorporate CBDPN, UNet, DeeplabV3, and DeeplabV3+ with the semi-supervised structure to train the biomarker prediction model. Note that in this case, the segmentation networks were used as the generator of the whole structure. By incorporating with the semi-supervised structure, CBDPN was demonstrated to be capable of predicting cellular biomarker distributions that are similar to the corresponding mIHC staining results (Figure 5). An example of an H&E-stained slide is shown in Figure 5A; the corresponding mIHC image and prediction generated by CBDPN are shown in Figure 5B,C, respectively. In Figure 5D, we compared results generated by CBDPN and other three conventional segmentation networks under the semi-supervised structure. The visualization of the feature maps obtained after softmax operation are illustrated in Figure S4. It can be seen that our method yielded cellular biomarker distribution prediction results that were similar to the results of mIHC staining analysis.

The quantified results of our test set (including 814 H&E patches with labels) are summarized in Table 2. As observed, our CBDPN also achieved optimal results in the semi-supervised experiments. The results of all performance indicators revealed that CBDPN consistently outperformed the other networks. Our semi-supervised strategy also proved to be effective in the biomarker distribution prediction task, as it increased accuracy by 2.9%, up to 90.4% in the case of CBDPN. Increases were also observed in the tests conducted using the other three networks. The semi-supervised mechanism enables unlabeled images being trained in a self-taught manner, so that more image data can be leveraged in the training phase.

#### 3.1.3. TCGA Dataset Validation Results

**Figure 6:**
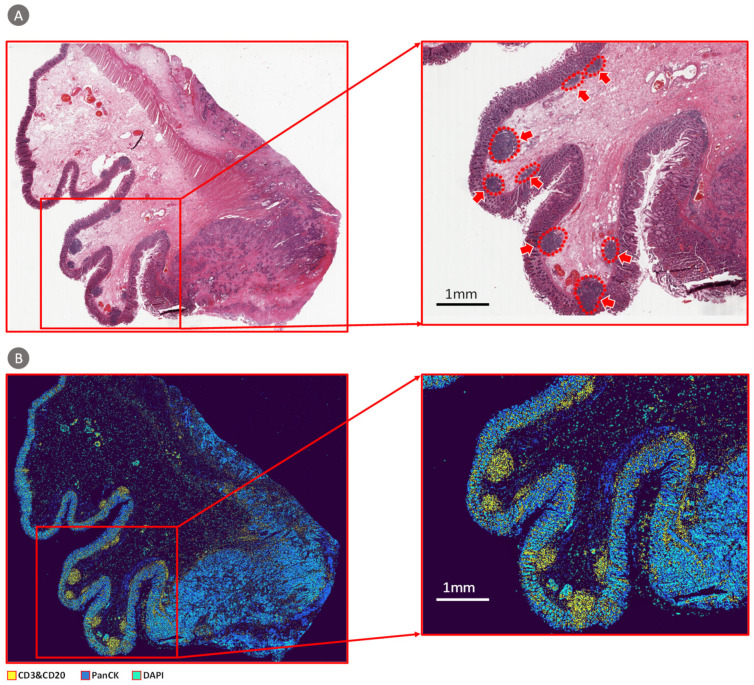
Visualization of the prediction result of a whole slide image (WSI) from the TCGA dataset. (**A**) H&E stained WSI (filename: TCGA-AZ-4616-01Z-00-DX1). The red arrows indicate pathologists confirmed tumor infiltrating lymphocytes (TILs) clusters. (**B**) Prediction result acquired using semi-supervised CBDPN.

**Figure 7:**
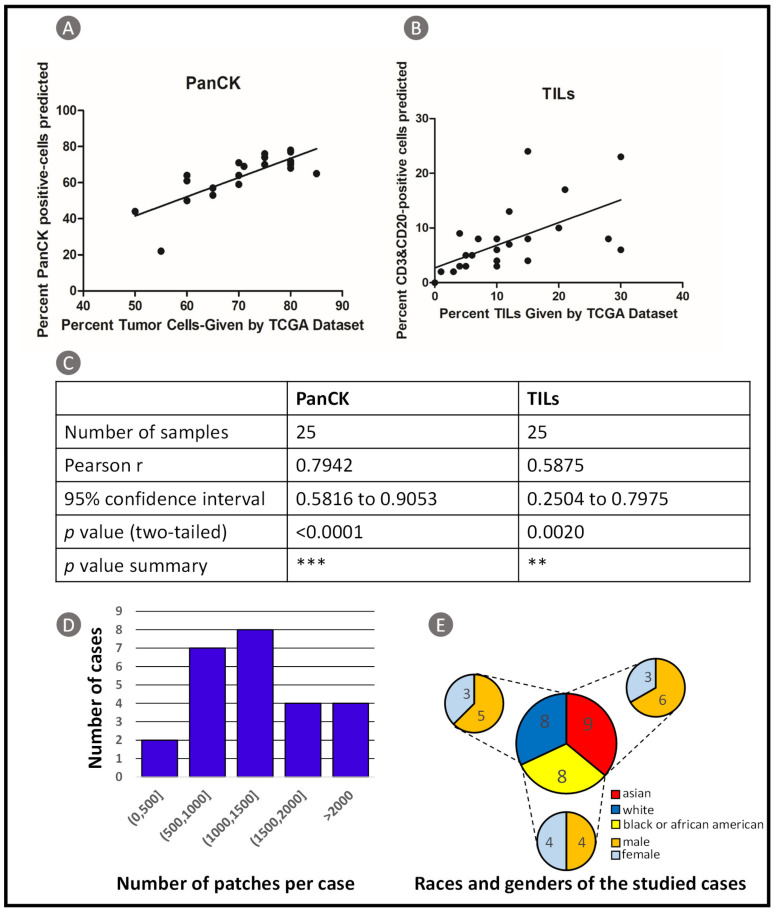
TCGA dataset validation results. (**A**) Scatter plot of the PanCK-positive cell proportion: results based on CBDPN versus results based on molecular information provided by TCGA. (**B**) Scatter plot of the TILs proportion: results based on CBDPN (Note that TILs were labeled by CD3 and CD20 in this study) versus results based on molecular information provided by TCGA. (**C**) Correlation analysis between results based on CBDPN and results based on TCGA information. (**D**) Distribution of the number of tiles per case. (**E**) Races and genders distribution of the cases used in this experiment.

To confirm the generalizability and robust of our model, we also used data of 25 patients from TCGA dataset for validation. A prediction result visualization of TCGA whole slide image (WSI) is shown in Figure 6. Board certified pathologists were asked to make judgements of the spatial distribution of TILs on the H&E WSI. It can be seen that TIL clusters predicted by our method is consistent with the judgment of pathologists. We compared proportions of cancer cells and TILs calculated based on CBDPN predictions and molecular information provided by TCGA. The details are illustrated in Figure 7. The scatter plot is shown in Figure 7A, *p* values analysis and Pearson correlation coefficients are shown in Figure 7B. Pearson correlation coefficients for PanCK and TILs experiments are 0.7942 and 0.5875, respectively, accompanied by significant *p* values. The results of the statistical analysis revealed agreement between the cell proportions calculated by the CBDPN and those determined based on the molecular information of TCGA dataset. The number of image patches of the WSIs used in this experiment is described in Figure 7C and races and genders distribution of these 25 patients is shown in Figure 7D. These results prove that our proposed biomarker prediction network has good generalizability and robustness across various tissue samples.

#### 3.1.4. CBDPN-Based Cell Quantification Analysis

PD-1, a critical immune checkpoint molecule, plays an important role in determining the TME immune status. In this study, we tested the ability of CBDPN to help quantify PD-1 expression in TME in the presence of anti-PD-1 immunofluorescence staining.

**Figure 8:**
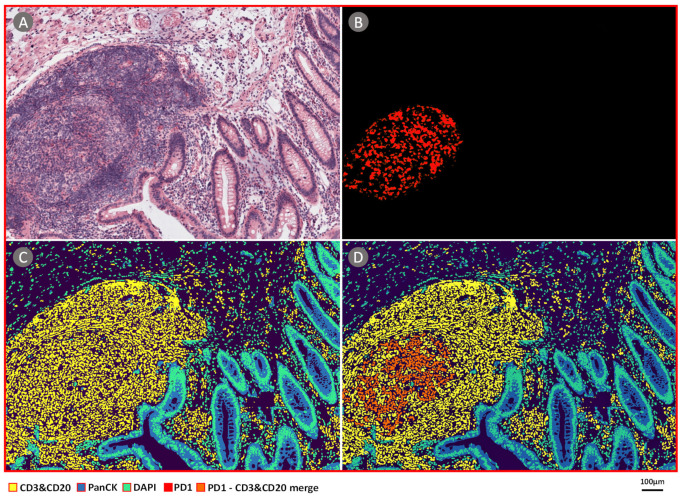
PD-1 distribution analysis with the ImmunoAIzer CBDPN. (**A**) H&E-stained colon cancer sample slide. (**B**) Anti-PD-1 immunofluorescence-stained image of the same tissue slide. (**C**) Cell-specific biomarker distribution image generated by CBDPN. (**D**) Merged image showing the stained PD-1-positive cells overlaid on our predicted cell-specific biomarker distribution image, the orange area represents PD-1-positive TILs.

**Figure 9:**
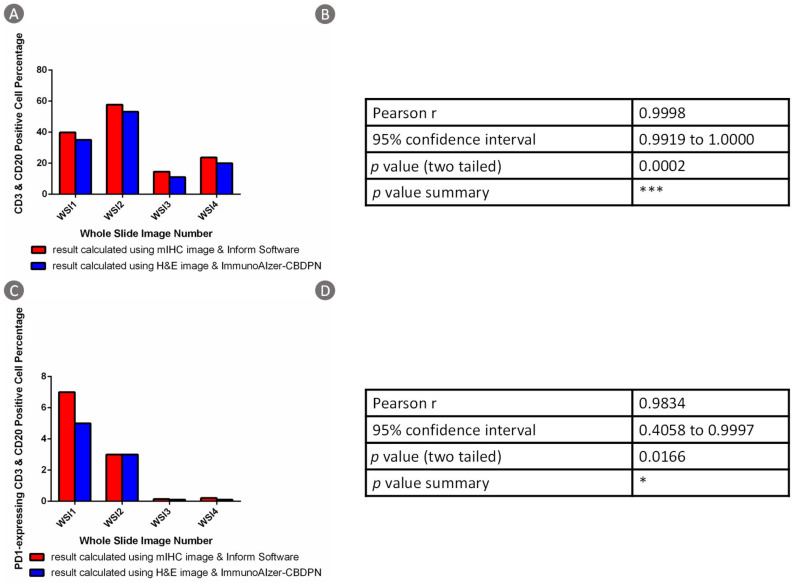
Comparative analysis of cell quantification based on semi-supervised CBDPN and commercial Inform Software (Version 2.4). (**A**) Bar chart comparison of CD3- and CD20-positive cell percentage calculated based on CBDPN and Inform software. (**B**) Correlation analysis of CD3- and CD20-positive cell percentage calculated based on CBDPN and Inform software. (**C**) Bar chart comparison of PD-1-expressing TILs percentage calculated based on CBDPN and Inform software. (**D**) Correlation analysis of PD-1-expressing TILs percentage calculated based on CBDPN and Inform software.

In total, all of the 94,662 H&E image patches of WSIs of four colon cancer patients from Peking University Cancer Hospital and Institute were analyzed by CBDPN in the cell quantification experiment. One sample image is shown in Figure 8. Figure 8A shows the H&E image and Figure 8B shows the anti-PD-1 immunofluorescence-stained image of the same tissue. The prediction result generated by CBPDN is shown in Figure 8C, and the merged image showing PD-1 expressing TILs is shown in Figure 8D. The sizes of the four WSIs are described in Table S1. We compared results obtained via our approach, and those obtained by applying the commercial Inform software to analyze the mIHC image of the same tissue. CD3 and CD20-positive cell percentage comparison results are described in Figure 9A,B. In addition, PD-1-expressing CD3 and CD20-positive cell percentage comparison results are described in Figure 9C,D. The results showed that most of the cells expressing PD-1 were TILs. Further, agreement can be seen between results based on CBDPN and Inform software, with Pearson correlation values as 0.9998 and 0.9834 for the two quantification experiments accompanied by highly significant *p* values. This further proves that our proposed method can help quantify TILs and PD-1-expressing TILs in TME accurately. Moreover, quantification using CBDPN was achieved at a lower cost and faster speed than conventional methods based on mIHC, and thus it can be beneficial for TME analysis. We believe that, given the ability of our network to quantify PD-1 expression, CBDPN could also be used to facilitate the quantification of other biomarkers in the presence of certain immunochemistry stains.

### 3.2. TGMDN for the Detection of Tumor Gene Mutations

#### 3.2.1. Detection of Tumor Gene Mutations from H&E Images

We next focused on predicting APC, KRAS, and TP53 gene mutations in the colon cancer tumor areas by using H&E image data as the input to a multilabel prediction network. We selected 446 patients in total, and the gene mutation status of the dataset is illustrated in Supplementary Figure S2. In total 44,534 H&E patches were used in the gene mutation detection experiment, of which 31,174 H&E image patches were included in the training set, 6680 H&E image patches in the validation set, and 6680 H&E image patches in the test set.

**Figure 10:**
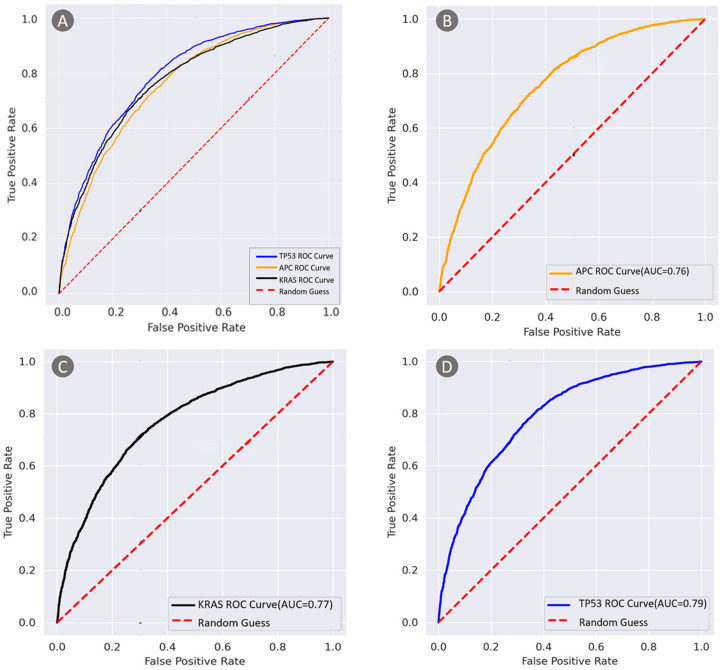
Receiver operating characteristic curves for tumor gene mutation detection network. (**A**) Receiver operating characteristic curve (ROC) and area under curve (AUC) value for adenomatous polyposis coli gene (APC), tumor protein P53 gene (TP53), and kirsten rat sarcoma viral oncogene (KRAS) mutations. (**B**) ROC curve and AUC value for APC mutation. (**C**) ROC curve and AUC value for KRAS mutation. (**D**) ROC curve and AUC value for TP53 mutation.

The receiver operating characteristic (ROC) curves are shown in Figure 10. The area-under-the-curve (AUC) values for *APC*, *TP53*, and *KRAS* were 0.76, 0.79, and 0.77, respectively, indicating that these three gene mutations were detectable in the tumor areas analyzed by TGMDN.

#### 3.2.2. Visualization of Network Features

**Figure 11:**
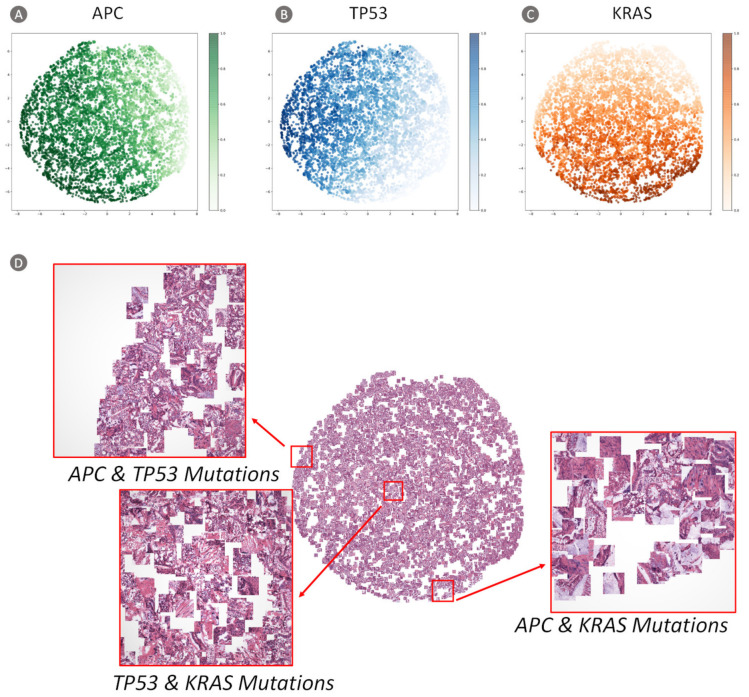
Two-dimensional visualization of TGMDN output obtained by implementing the t-SNE Algorithm. (**A**) Cluster result of *APC* mutation probability generated by the TGMDN. (**B**) Cluster result of *TP53* mutation probability generated by the TGMDN. (**C**) Cluster result of *KRAS* mutation probability generated by the TGMDN. (**D**) Patch-embedded t-SNE representation with magnifications showing specific mutations that were detected based on the H&E image data from test set that were obtained from the TCGA Colon adenocarcinoma (COAD) project.

The cluster results based on t-SNE are shown in Figure 11A–C, with each dot representing an image patch and its color intensity indicating the probability of our model to predict the tumor gene mutation. The H&E patch-embedded representation (Figure 11D) can be used to visualize patches predicted to have similar mutations, and thus possess the potential to help reveal the associations of gene mutations. In Figure 11D, the clusters in the top-left enlargement comprise patches constituting APC and TP53 gene mutations. The clusters in the center reveal the co-existence of TP53 and KRAS gene mutations. Finally, the clusters in the bottom-right enlargement comprise patches that confirm the co-existence of APC and KRAS gene mutations. These findings suggest that our ImmunoAIzer-TGMDN can be used to identify such genotype–phenotype association in colon cancer.

## 4. Discussion

In this work, we proposed a computational framework, termed ImmunoAIzer, which takes H&E image patches as inputs and consists of two components: (1) CBDPN to make predictions of the spatial distribution of CD3, CD20, PanCK, and DAPI in TME, and (2) TGMDN to detect APC, TP53, and KRAS mutations.

Our study demonstrates that CBDPN can make predictions of distributions of CD3, CD20, PanCK, and DAPI with an accuracy of 90.4%, demonstrating that it can be used to reveal the spatial distributions of TILs and cancer cells in TME, and is therefore of significant value as a tool for TME analysis. To maximize the utility of our proposed ImmunoAIzer framework as a tool for clinical application, we incorporated a tumor gene detection network. We used TGMDN to predict the mutation status of three of the most common mutations in TCGA COAD project, namely, APC, TP53, and KRAS. The predicted AUC values for these three mutations were 0.76, 0.79, and 0.77, respectively; these results demonstrate the feasibility of TGMDN as a tool to predict tumor gene mutation status. In general, our approach demonstrates that DL-based methods can be reasonably applied to predict the spatial distribution of TILs and cancer cells in TME, meanwhile to detect gene mutations of colon cancer patients based on histological image data. This can be highly beneficial for the guidance of immunotherapy and prognoses.

The spatial distribution of TILs and cancer cells in TME is of crucial importance for the guidance of immunotherapy and prognosis. The acquisition of such information often requires mIHC staining, which can be time- and cost-consuming, and the interpretation of the staining result can be compromised by individual subjectivity; this highlights the necessity for the development of a standardized methodology to provide pathologists with comprehensive references for TME analysis. Despite advances in DL-based computational methods, current fully supervised DL-based methods often require large amount of annotated image data to achieve good generalization ability and robustness. However, it can be extremely time consuming to annotate each cell on histological image in practice. Thus, our proposed computational framework ImmunoAIzer includes a semi-supervised CBDPN to leverage both labeled and unlabeled image data to make accurate and robust predictions of spatial distribution of TILs and cancer cells in TME, which highly reduces the burden of annotation work and makes it more suitable for clinical use. The increasing data size can contribute to the robustness of the model and improve the generalization performance as well as prediction accuracy on the test set. These account for the improvement of models when switching from fully to semi-supervised strategy. Both CBDPN and UNet adopted the idea of multi-scale feature fusion, which makes both of them superior in pathological image data processing tasks. However, CBDPN not only conducts a feature concatenation process between the encoder and decoder, but also used a series of multi-resolution convolutional modules within the encoder structure to enhance the feature extraction performance, which made the performance advantage of CBPDN more obvious when the data size is increased. The results demonstrate the effectiveness of the semi-supervised mechanism to make full use of information encoded in the unlabeled image data to boost prediction performance. To the best of our knowledge, ImmunoAIzer is the first DL-based computational framework that utilizes a semi-supervised learning strategy to make predictions of the spatial distribution of immune cells and cancer cells in TME. Moreover, the incorporation of anti-PD-1 immunofluorescence staining image enables the CBDPN to be used to aid quantification of the PD-1-positive cells in TME. The implementation of CBDPN offers significant time and cost savings compared to stain-based methods. These findings demonstrate that the ImmunoAIzer can serve as an auxiliary tool for providing guidance for TME analysis.

Meanwhile, the results of incorporating a gene mutation detection network that enables detections about APC, TP53, and KRAS mutations revealed the potential of computational methods for implementation in gene mutation detection tasks. The t-SNE cluster results provided further insight into the associations between APC, TP53, and KRAS mutations; this type of information can be well utilized in tumor gene mutation association studies. Thus, this study is also useful as a reference for tumor gene mutation analysis, and as a general introduction to computational methods as a valuable tool for clinical usage.

The necessity of a tool with the ability to increase the amount of useful information that can be acquired from histological images is increasingly being acknowledged as critical for therapeutic optimization and patient selection [44]. ImmunoAIzer is reproducible and provides an efficient tool for the cellular biomarker distribution prediction and tumor gene mutation detection. Moreover, the dataset consists of H&E images with pixel-level labels which can be used to facilitate development of other DL-based computational tools.

It is worth mentioning that our model was initially trained on H&E data from colon cancer patients, the performance of the model on other cancer types has yet to be validated. However, we believe this methodology is generalizable and could be used to analyze data of other cancer types. In our future work, we will extend the application of ImmunoAIzer to other cancer types and immune biomarkers. For the network structure, the attention mechanism [45] can be further incorporated into the convolution process to optimize the model and thus improve the prediction accuracy. We hope that by applying our computational framework to recognize a wider range of pathological features in TME, we will be able to gain more insights and offer more guidance for therapeutic optimization and prognoses.

## 5. Conclusions

ImmunoAIzer can utilize H&E images to provide comprehensive information about spatial distribution of TILs and cancer cells in TME, meanwhile detect tumor gene mutations as APC, KRAS, and TP53. Our work demonstrates the potential of DL-based tools to aid pathologists in clinical use.

## Figures and Tables

**Table 1:** Fully-supervised learning comparisons of the four networks on the test set.

Network	Accuracy	Precision	Recall	Dice	IoU
UNet	0.873	0.799	**0.871**	0.825	0.714
DeepLab V3	0.856	0.776	0.836	0.797	0.677
DeepLab V3+	0.863	0.778	0.858	0.805	0.690
CBPDN	**0.875**	**0.806**	0.865	**0.827**	**0.717**

The bold font indicates the best result.

**Table 2:** Semi-supervised learning comparisons of the four networks on the test set.

Network	Accuracy	Precision	Recall	Dice	IoU
UNet	0.891	0.804	0.863	0.824	0.711
DeepLab V3	0.868	0.789	0.859	0.813	0.701
DeepLab V3+	0.872	0.803	0.865	0.823	0.712
CBPDN	**0.904**	**0.854**	**0.901**	**0.872**	**0.788**

The bold font indicates the best result.

## Data Availability

The labeled data used for CBDPN can be downloaded from: https://data.mendeley.com/datasets/g52z2hmxrx/1 (accessed on 20 February 2021). The original scanning WSIs can be accessed at: https://data.mendeley.com/datasets/6rkw48wspj/draft?a=a324f843-526f-4dac-a8e5-c26ca52c58f0 (accessed on 20 February 2021). The source code can be accessed at https://github.com/BianChang/ImmunoAIzer (accessed on 20 February 2021). The unlabeled original WSIs used for CBDPN and TGMDN can be downloaded from the TCGA dataset: https://portal.gdc.cancer.gov/ (accessed on 8 February 2021).

## Supplementary Materials

The following are available online at https://www.mdpi.com/article/10.3390/cancers13071659/s1, Table S1: Scanned image sizes of the four H&E whole slide images (WSIs) for cell quantification experiment. Figure S1: Construction pipeline of the dataset with H&E images and corresponding pixel-level labels. Figure S2: Distribution of most frequently mutated genes of the 446 cases from TCGA COAD project. Figure S3: Internal structure of CBPDN blocks. Figure S4: Feature maps obtained after softmax operation of CBDPN.

## Appendix A

In this appendix, we will describe our staining and scanning protocols.

We acquired tissue samples from eight colon cancer patients from the Key Laboratory of Carcinogenesis and Translational Research (Ministry of Education), Peking University Cancer Hospital and Institute. The mIHC images were obtained using a PANO 7-plex IHC kit (Panovue, Beijing, China). We applied different colored chromogens including CD3 and CD20 to label TILs [26], PanCK to label cancer cells and DAPI to label other cell nuclei. Then, performed horseradish peroxidase-conjugated secondary antibody incubation and tyramide signal amplification. A microwave heat treatment was applied to the samples after each tumor-specific antigen operation. DAPI (Sigma-Aldrich, Shanghai, China) was used to stain nuclei. After the mIHC slides were scanned and the mIHC stain was verified, the sample slides were treated using microwave and then bleached using sterile water. The slides were further processed for H&E staining using an H&E staining kit (Solarbio, Beijing, China).

In order to acquire a clear view of the structural details of the pathological tissue, the H&E- and mIHC-stained slides were scanned at 40× magnification using a high-resolution Mantra system scanner (PerkinElmer, Waltham, MA, USA).

## Appendix B

In this appendix, we will describe the image processing procedure during constructing the dataset.

The registration procedure was performed as follows:

1. Coarse block matching: Generally, the original WSIs are extremely large in size and thus difficult to process in their entirety owing to RAM limitations. To address this problem, we randomly selected 15 candidate blocks (~5000 × ~4000 pixels) in each mIHC staining WSI. Then, a normalized correlation matrix was calculated by correlating each of the ~5000 × ~4000-pixel blocks with the corresponding block extracted from the whole-slide grayscale H&E image of the same size. The block with the highest correlation score was considered to be the coarsely matched H&E block for the two staining blocks.
2. Global registration: After acquiring coarsely matched block pairs. A global registration step was carried out to correct the slight rotation angle. We extracted feature vectors (descriptors) and their corresponding locations from the block pairs; we then matched the features using the descriptors [46]. Next, the M-estimator sample consensus algorithm was used to calculate the transformation matrix [47]. After the rotation was applied, the images were cropped, removing 50 pixels on each side to eliminate the undefined areas that resulted from the rotation.
3. Elastic registration: After Step 2, an elastic registration between H&E image blocks and globally registered mIHC image blocks was conducted by applying a diffeomorphic demons algorithm [48] to correct the distortions induced by warping and various aberrations.

After the registration procedure was completed, the registered block pairs were tiled into patches of 512 × 512 pixels and pixel-level annotated by 3 board certified pathologists. Then, all of the H&E image patches were processed to apply stain intensity normalization [27,28]. Finally, an image augmentation step was carried out to enrich the dataset and improve generalizability. We applied image augmentation to all H&E patches. The transformations that were applied to our original H&E patches included the Gaussian blur, flipping, rotations, and Gaussian noise. The augmentation code implemented in this study was taken from https://github.com/codebox/image_augmentor (accessed on 2 February 2021).

## References

[1] Balkwill F.R., Capasso M., Hagemann T. (2012). The tumor microenvironment at a glance. J. Cell Sci..

[2] Hanahan D., Coussens L.M. (2012). Accessories to the crime: Functions of cells recruited to the tumor microenvironment. Cancer Cell.

[3] Whiteside T.J.O. (2008). The tumor microenvironment and its role in promoting tumor growth. Oncogene.

[4] Cristescu R., Mogg R., Ayers M., Albright A., Murphy E., Yearley J., Sher X., Liu X.Q., Lu H.C., Nebozhyn M. (2018). Pan-tumor genomic biomarkers for PD-1 checkpoint blockade-based immunotherapy. Science.

[5] Du Y., Jin Y.H., Sun W., Fang J.J., Zheng J.J., Tian J. (2019). Advances in molecular imaging of immune checkpoint targets in malignancies: Current and future prospect. Eur. Radiol..

[6] Du Y., Qi Y.F., Jin Z.G., Tian J. (2019). Noninvasive imaging in cancer immunotherapy: The way to precision medicine. Cancer Lett..

[7] Rosenberg S.A., Spiess P., Lafreniere R. (1986). A New Approach To the Adoptive Immunotherapy of Cancer with Tumor-Infiltrating Lymphocytes. Science.

[8] Roth A.D., Delorenzi M., Tejpar S., Yan P., Klingbiel D., Fiocca R., d’Ario G., Cisar L., Labianca R., Cunningham D. (2012). Integrated Analysis of Molecular and Clinical Prognostic Factors in Stage II/III Colon Cancer. J. Natl. Cancer Inst..

[9] Markowitz S.D., Dawson D.M., Willis J., Willson J.K.V. (2002). Focus on colon cancer. Cancer Cell.

[10] Westra J.L., Schaapveld M., Hollema H., de Boer J.P., Kraak M.M.J., de Jong D., ter Elst A., Mulder N.H., Buys C.H.C.M., Hofstra R.M.W. (2005). Determination of TP53 mutation is more relevant than microsatellite instability status for the prediction of disease-free survival in adjuvant-treated stage III colon cancer patients. J. Clin. Oncol..

[11] Liao W.T., Overman M.J., Boutin A.T., Shang X.Y., Zhao D., Dey P., Li J.X., Wang G.C., Lan Z.D., Li J. (2019). KRAS-IRF2 Axis Drives Immune Suppression and Immune Therapy Resistance in Colorectal Cancer. Cancer Cell.

[12] Yarchoan M., Hopkins A., Jaffee E.M. (2017). Tumor Mutational Burden and Response Rate to PD-1 Inhibition. N. Engl. J. Med..

[13] Kalra J., Baker J., Kalyuzhny A.E. (2017). Multiplex Immunohistochemistry for Mapping the Tumor Microenvironment. Signal Transduction Immunohistochemistry: Methods and Protocols.

[14] Stack E.C., Wang C.C., Roman K.A., Hoyt C.C. (2014). Multiplexed immunohistochemistry, imaging, and quantitation: A review, with an assessment of Tyramide signal amplification, multispectral imaging and multiplex analysis. Methods.

[15] Tsujikawa T., Kumar S., Borkar R.N., Azimi V., Thibault G., Chang Y.H., Balter A., Kawashima R., Choe G., Sauer D. (2017). Quantitative Multiplex Immunohistochemistry Reveals Myeloid-Inflamed Tumor-Immune Complexity Associated with Poor Prognosis. Cell Rep..

[16] Blom S., Paavolainen L., Bychkov D., Turkki R., Maki-Teeri P., Hemmes A., Valimaki K., Lundin J., Kallioniemi O., Pellinen T. (2017). Systems pathology by multiplexed immunohistochemistry and whole-slide digital image analysis. Sci. Rep..

[17] Ionescu G.V., Fergie M., Berks M., Harkness E.F., Hulleman J., Brentnall A.R., Cuzick J., Evans D.G., Astley S.M. (2019). Prediction of reader estimates of mammographic density using convolutional neural networks. J. Med. Imaging.

[18] Campanella G., Hanna M.G., Geneslaw L., Miraflor A., Silva V.W.K., Busam K.J., Brogi E., Reuter V.E., Klimstra D.S., Fuchs T.J. (2019). Clinical-grade computational pathology using weakly supervised deep learning on whole slide images. Nat. Med..

[19] Rivenson Y., Wang H.D., Wei Z.S., de Haan K., Zhang Y.B., Wu Y.C., Gunaydin H., Zuckerman J.E., Chong T., Sisk A.E. (2019). Virtual histological staining of unlabelled tissue-autofluorescence images via deep learning. Nat. Biomed. Eng..

[20] Saltz J., Gupta R., Hou L., Kurc T., Singh P., Nguyen V., Samaras D., Shroyer K.R., Zhao T.H., Batiste R. (2018). Spatial Organization and Molecular Correlation of Tumor-Infiltrating Lymphocytes Using Deep Learning on Pathology Images. Cell Rep..

[21] Christiansen E.M., Yang S.J., Ando D.M., Javaherian A., Skibinski G., Lipnick S., Mount E., O’Neil A., Shah K., Lee A.K. (2018). In Silico Labeling: Predicting Fluorescent Labels in Unlabeled Images. Cell.

[22] Burlingame E.A., Margolin A.A., Gray J.W., Chang Y.H. (2018). SHIFT: Speedy histopathological-to-immunofluorescent translation of whole slide images using conditional generative adversarial networks. Medical Imaging 2018: Digital Pathology.

[23] Coudray N., Ocampo P.S., Sakellaropoulos T., Narula N., Snuderl M., Fenyö D., Moreira A.L., Razavian N., Tsirigos A.J.N.m. (2018). Classification and mutation prediction from non–small cell lung cancer histopathology images using deep learning. Nat. Med..

[24] Litjens G., Kooi T., Bejnordi B.E., Setio A.A.A., Ciompi F., Ghafoorian M., van der Laak J.A.W.M., van Ginneken B., Sanchez C.I. (2017). A survey on deep learning in medical image analysis. Med. Image Anal..

[25] Chapelle O., Scholkopf B., Zien A. (2009). Semi-supervised learning (chapelle, o. et al., eds.; 2006) [book reviews]. IEEE Trans. Neural Netw..

[26] Brown J.R., Wimberly H., Lannin D.R., Nixon C., Rimm D.L., Bossuyt V. (2014). Multiplexed Quantitative Analysis of CD3, CD8, and CD20 Predicts Response to Neoadjuvant Chemotherapy in Breast Cancer. Clin. Cancer Res..

[27] Macenko M., Niethammer M., Marron J.S., Borland D., Woosley J.T., Guan X.J., Schmitt C., Thomas N.E. A Method for Normalizing Histology Slides for Quantitative Analysis. Proceedings of the 2009 IEEE International Symposium on Biomedical Imaging: From Nano To Macro.

[28] Vahadane A., Peng T.Y., Sethi A., Albarqouni S., Wang L.C., Baust M., Steiger K., Schlitter A.M., Esposito I., Navab N. (2016). Structure-Preserving Color Normalization and Sparse Stain Separation for Histological Images. IEEE T Med. Imaging.

[29] Hung W.C., Tsai Y.H., Liou Y.T., Lin Y.Y., Yang M.H. Adversarial Learning for Semi-Supervised Semantic Segmentation. Proceedings of the British Machine Vision Conference.

[30] He K., Zhang X., Ren S., Sun J. Deep residual learning for image recognition. Proceedings of the IEEE Conference on Computer Vision and Pattern Recognition.

[31] Szegedy C., Vanhoucke V., Ioffe S., Shlens J., Wojna Z. Rethinking the Inception Architecture for Computer Vision. Proceedings of the IEEE Conference on Computer Vision and Pattern Recognition.

[32] Ronneberger O., Fischer P., Brox T. U-Net: Convolutional Networks for Biomedical Image Segmentation. Proceedings of the International Conference on Medical Image Computing and Computer-Assisted Intervention.

[33] Chen L.-C., Papandreou G., Schroff F., Adam H. (2017). Rethinking Atrous Convolution for Semantic Image Segmentation. arXiv.

[34] Chen L.-C., Zhu Y., Papandreou G., Schroff F., Adam H. Encoder-decoder with atrous separable convolution for semantic image segmentation. Proceedings of the European Conference on Computer Vision (ECCV).

[35] Topalian S.L., Hodi F.S., Brahmer J.R., Gettinger S.N., Smith D.C., McDermott D.F., Powderly J.D., Carvajal R.D., Sosman J.A., Atkins M.B. (2012). Safety, Activity, and Immune Correlates of Anti-PD-1 Antibody in Cancer. N. Engl. J. Med..

[36] Wyss J., Dislich B., Koelzer V.H., Galvan J.A., Dawson H., Hadrich M., Inderbitzin D., Lugli A., Zlobec I., Berger M.D. (2019). Stromal PD-1/PD-L1 Expression Predicts Outcome in Colon Cancer Patients. Clin. Colorectal Cancer.

[37] Sehdev A., Cramer H.M., Ibrahim A.A., Younger A.E., O’Neil B.H. (2016). Pathological Complete Response with Anti-PD-1 Therapy in a Patient with Microsatellite Instable High, BRAF Mutant Metastatic Colon Cancer: A Case Report and Review of Literature. Discov. Med..

[38] Schindelin J., Arganda-Carreras I., Frise E., Kaynig V., Longair M., Pietzsch T., Preibisch S., Rueden C., Saalfeld S., Schmid B. (2012). Fiji: An open-source platform for biological-image analysis. Nat. Methods.

[39] Kramer A.S., Latham B., Diepeveen L.A., Mou L.J., Laurent G.J., Elsegood C., Ochoa-Callejero L., Yeoh G.C. (2018). InForm software: A semi-automated research tool to identify presumptive human hepatic progenitor cells, and other histological features of pathological significance. Sci. Rep..

[40] Ma N., Zhang X., Zheng H.-T., Sun J. ShuffleNet V2: Practical Guidelines for Efficient CNN Architecture Design. Proceedings of the European Conference on Computer Vision.

[41] Kather J.N., Heij L.R., Grabsch H.I., Loeffler C., Echle A., Muti H.S., Krause J., Niehues J.M., Sommer K.A., Bankhead P.J.N.C. (2020). Pan-cancer image-based detection of clinically actionable genetic alterations. Nat. Cancer.

[42] Van der Maaten L., Hinton G. (2008). Visualizing data using t-SNE. J. Mach. Learn. Res..

[43] Paszke A., Gross S., Chintala S., Chanan G., Yang E., DeVito Z., Lin Z., Desmaison A., Antiga L., Lerer A. Automatic Differentiation in Pytorch. https://openreview.net/forum?id=BJJsrmfCZ.

[44] Gurcan M.N., Boucheron L.E., Can A., Madabhushi A., Rajpoot N.M., Yener B. (2009). Histopathological image analysis: A review. IEEE Rev. Biomed. Eng..

[45] Chen L.C., Yang Y., Wang J., Xu W., Yuille A.L. Attention to Scale: Scale-aware Semantic Image Segmentation. Proceedings of the IEEE Conference on Computer Vision and Pattern Recognition.

[46] Lowe D.G. (2004). Distinctive image features from scale-invariant keypoints. Int. J. Comput. Vis..

[47] Torr P.H.S., Zisserman A. (2000). MLESAC: A new robust estimator with application to estimating image geometry. Comput. Vis. Image Underst..

[48] Vercauteren T., Pennec X., Perchant A., Ayache N. (2009). Diffeomorphic demons: Efficient non-parametric image registration. Neuroimage.

