# Supplementary material for: ImmunoAIzer: A Deep Learning-Based Computational Framework to Characterize Cell Distribution and Gene Mutation in Tumor Microenvironment
Source: Cancers (Basel). 2021 Apr 1;13(7):1659. doi: 10.3390/cancers13071659 (PMC8036970; doi:10.3390/cancers13071659)
Supplement: Supplementary file 1 [file cancers-13-01659-s001.pdf]

cancers MDPI

Supplementary

ImmunoAIzer: A Deep Learning-Based Computational Framework to Characterize Cell Distribution and Gene Mutation in Tumor Microenvironment

Chang Bian, Yu Wang, Zhihao Lu, Yu An, Hanfan Wang, Lingxin Kong, Yang Du* and Jie Tian*

Table S1. Scanned image sizes of the four H&E Whole Slide Images (WSIs) for cell quantification experiment.

Image Information	WSI1	WSI2	WSI3	WSI4
Image Size	13.43mm × 19.42mm	15.83mm × 18.34mm	19.18mm × 25.54mm	19.66mm × 26.26mm
Resolution	0.25µm/pixel	0.25µm/pixel	0.25µm/pixel	0.25µm/pixel
Size in Pixels	53760 × 77760	63363 × 73440	76800 × 102240	78720 × 105120

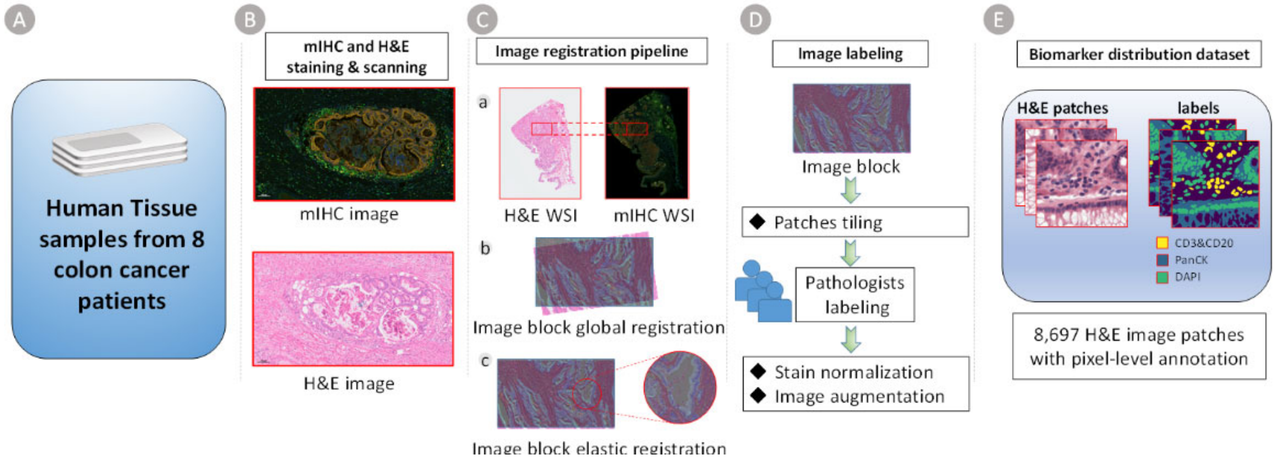

Figure S1. Construction pipeline of the dataset with H&E images and corresponding pixel-level labels. (**A**) Human tissue samples were acquired from eight colon cancer patients. (**B**) Tissue samples were stained and scanned to acquire H&E and mIHC images. (**C**) A series of registration procedures, including image block matching, global registration, and elastic registration were conducted to ensure pixel-wise registration of the mIHC images and H&E images for annotation. (**D**) Registered image blocks were tiled into 512 × 512-pixel patches and sent to pathologists for annotation, and then we conducted stain normalization and image augmentation. (**E**) A cellular biomarker distribution dataset, including H&E image patches and corresponding biomarker distribution label masks, was constructed.

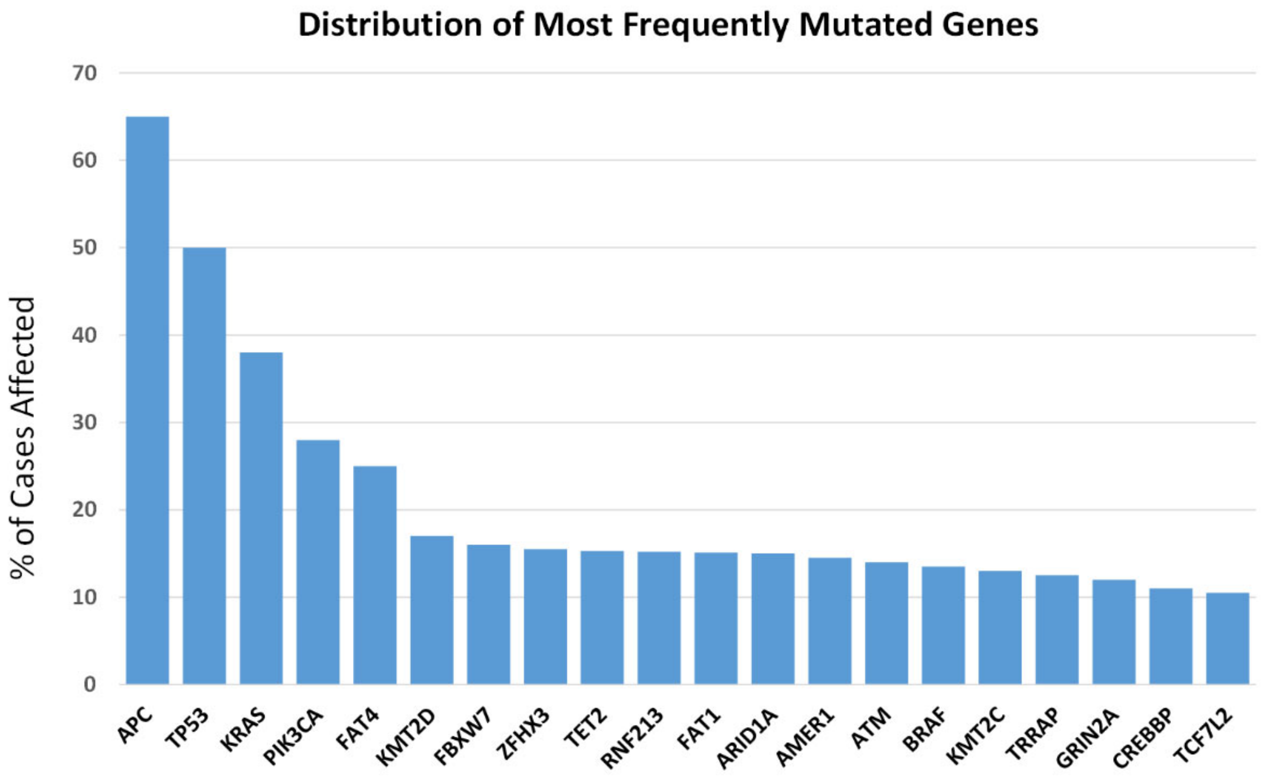

Figure S2. Distribution of most frequently mutated genes of the 446 cases from TCGA COAD project.

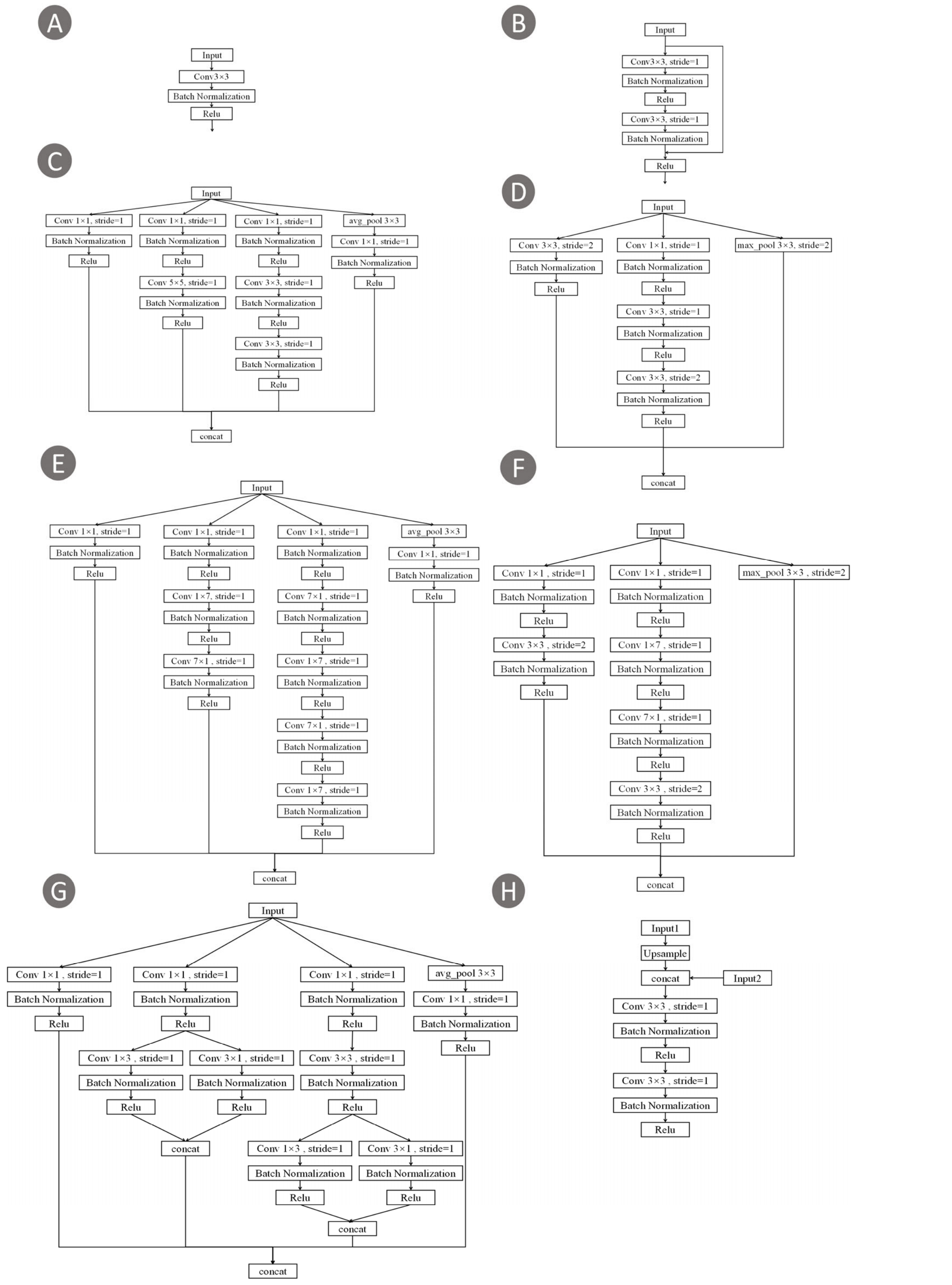

Figure S3. Internal structure of CBPDN blocks. (**A**) Convolutional block structure. (B) Residual block structure. (**C**) Inception A structure. (**D**) Inception B structure. (**E**) Inception C structure. (**F**) Inception D structure. (**G**) Inception E structure. (**H**) Upsample block structure.

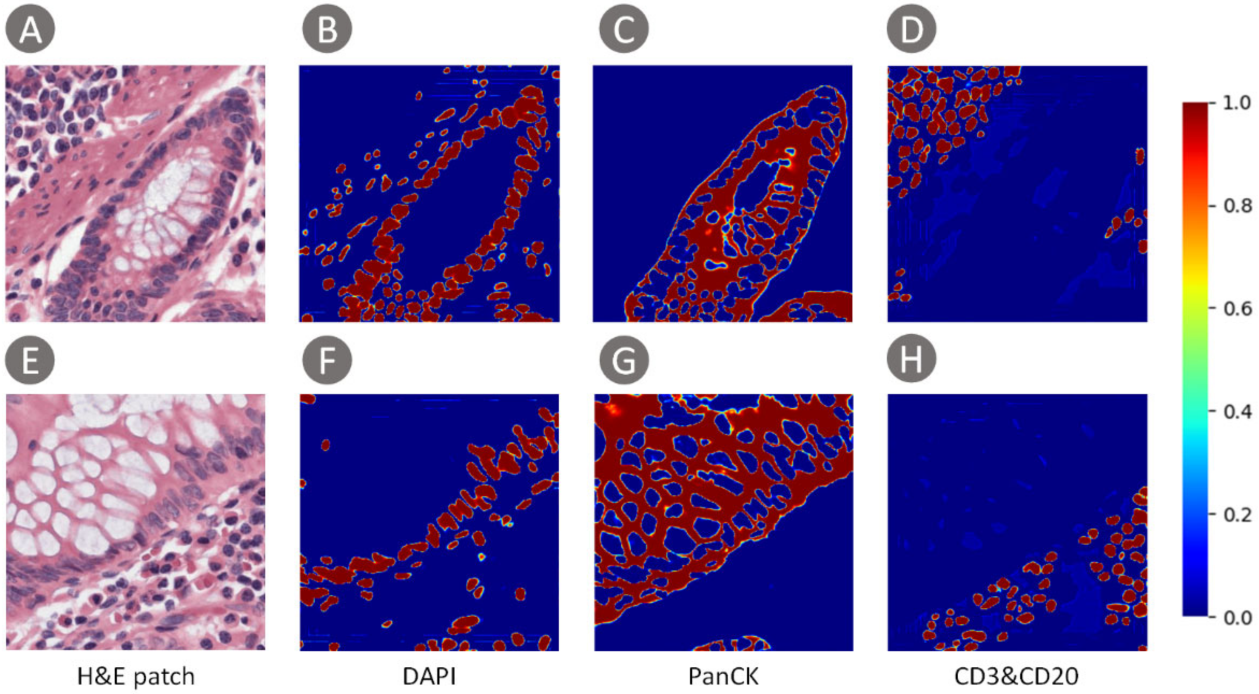

Figure S4. Feature maps obtained after softmax operation of CBDPN. The intensity of the color bar indicates the probability of the corresponding pixel being predicted. (**A**)&(**E**) H&E image patch, (**B**)&(**F**) DAPI, (**C**)&(**G**) PanCK, (**D**)&(**H**) CD3 and CD20.
